# Impact of interleukin-6 gene polymorphisms and its interaction with obesity on osteoporosis risk in Chinese postmenopausal women

**DOI:** 10.1186/s12199-019-0803-y

**Published:** 2019-07-13

**Authors:** Ya-Feng Ji, Xuesheng Jiang, Wei Li, Xingtao Ge

**Affiliations:** 1 Department of Orthopaedic Surgery, Huzhou Central Hospital, 198 Hongqi Road, Huzhou, 313000 Zhejiang Province China; 2 Department of Orthopedics 1, Rizhao People’s Hospital, Rizhao, 276800 Shandong China

**Keywords:** Osteoporosis, IL- 6, Polymorphism, Obesity, Interaction

## Abstract

**Aims:**

To investigate the association of four single-nucleotide polymorphisms (SNPs) of the IL-6 gene with osteoporosis (OST) susceptibility.

**Methods:**

PCR restriction fragment length polymorphism (PCR–RFLP) was carried out for SNPs detection. Generalized multifactor dimensionality reduction (GMDR) model and logistic regression model were used to examine the interaction between SNP and obesity on OST.

**Results:**

Logistic regression model revealed that G allele of rs1800796 and the T allele of rs2069849 were associated with increased OST risk, compared to those with wild genotype. However, no significant correlations were found when analyzing the association of rs1800795 and rs1554606 with OST risk. GMDR analysis suggested that the interaction model composed of the rs1800796 and obesity was the best model with statistical significance (*P* value from sign test [*P*_sign_] = 0.012), indicating a potential gene–environment interaction between rs1800796 and obesity. Overall, the two-locus models had a cross-validation consistency of 10/10 and had the testing accuracy of 0.641. We also conducted stratified analysis for rs1800796 genotype and obesity, and found that obese subjects with CG or GG genotype have the highest OST risk, compared to subjects with CC genotype, and normal BMI OR (95% CI) = 2.21 (1.52–3.49), after adjustment for age, smoke, and alcohol consumption status.

**Conclusions:**

Our results suggested that the C allele of *rs1800796* and the C allele of *rs2069849* of IL-6 gene interaction between *rs1800796* and abdominal obesity were all associated with increased OST risk.

## Introduction

Osteoporosis (OST) is a common disorder characterized by decreased bone mineral density (BMD), and it can increase the risk of fractures and worsen the quality of life of the aging population worldwide and featured by decreased bone mineral density (BMD) [1, 2]. According to reports, there are currently 83.9 million people in this age group in China, and this number by 2050 will increase to 212 million by 2050 [3]. BMD is affected by both environmental and genetic factors. However, although the environmental factors [4], especially diet and physical activity, affect BMD and menopausal women, the significant differences between BMD indices have their source in the genetic factors [5].

The IL-6 gene is located on human chromosome 7p21. Several polymorphisms in the IL-6 gene have been reported to have associations with carcinogenesis [6] and essential hypertension [7]. A recent study also demonstrated associations between single-nucleotide polymorphisms (SNPs) in the IL-6 genes and low BMD and OST in elderly people [8, 9]. However, these previous studies have not led to decisive conclusions. From the point of view of genetics, osteoporosis is a multifactorial disease, resulting from the activity of a variety of independent genes, as well as gene–gene and gene–environment interactions [10, 11]. Some environmental risk factors for OST were suggested, such as obesity [12, 13], smoking [14], alcohol drinking [15], and so on, which were studied in different populations. However, till now, no study focused on the impact of IL-6 gene–environment interaction on OST risk in the Chinese population. So the aim of this study was to investigate the association of four SNPs within the IL-6 gene and OST risk, and the impact of additional gene–environment interaction on OST based on Chinese postmenopausal females.

## Materials and methods

### Subjects

In this study, a total of 1524 Chinese post-menopausal women were selected from March 2010 to June 2016 in our hospital, including 758 patients with osteoporosis and 766 normal subjects. The primary post-menopausal osteoporosis is diagnosed by the doctor. Individuals having medical past or present history affecting bone metabolism, or individuals taking drugs that may affect bone metabolism were excluded from the investigation. Individuals in the control group did not have a family history of osteoporosis and matched the case based on age (± 3 years) and nearly 1:1. The collected blood samples were used to genotype polymorphisms from each participant. The informed consent was signed from everyone prior to participating in the study.

### Genomic DNA extraction and SNPs

Four tag-SNPs, comprising rs1800796, rs1800795, rs2069849, and rs1554606 were studied. Genomic DNA was isolated using EDTA-treated whole blood according to the manufacturer’s instructions of the DNA Blood Mini Kit (Qiagen, Hilden, Germany) and was stored at − 20 °C until use. PCR restriction fragment length polymorphism (PCR–RFLP) was carried out for SNPs detection and the primers for four SNPs were shown in Table 1. A 25 μl of the reaction volume in triplicate contains the Mastermix with dNTPs mix, reaction buffer, and Taq DNA polymerase (Shanghai, China): 0.5 μM of each primer, 2 μl of cDNA, 9 μl of 2.5× Mastermix, and 8.5 μl distilled water. After initial denaturation at 94 °C for 5 min, 40 cycles of PCR were performed for 30 s at 94 °C, for 45 s at 52.8 °C, and for 1 min at 72 °C, with a final extension at 72 °C for 7 min. Then, 8 μL of PCR product was digested with restriction endonuclease (Table 1) for 4 h at 37 °C. The digestion products were separated by electrophoresis on a 3% agarose gel and visualized with ethidium bromide. At lase, about 1/10 of the original specimens were assayed, and there was no difference between the confirmed genotypes by replica assays.

**Table 1:** Description of 4 SNPs within IL-6 gene and the primers designed

SNPs	Chromosome	Functional consequence	Restriction endonuclease	Alleles	Primer (5′→3′)
rs1800795-174G/C	7:22727026	Intron variant, upstream variant 2KB	*SfaNI*	G/ C	F: 5′-TGACTTCAGCTTTACTCTTGT-3′ R: 5′-CTGATTGGAAACCTTATTAAG-3′
rs1800796-572C/G	7:22726627	Intron variant, nc transcript variant, upstream variant 2KB	*BSrBI*	C/ G	F: 5′-AGATTCCAAGGGTCACTTG-3′ R: 5′-AGAAGCAGAACCACTCTTC-3′
rs1554606 185C/A	7:22729088	Intron variant, upstream variant 2KB	*EcoNI*	C/ A	F: 5′-ACTCATACATCTGGCCGTTG-3′ R: 5′-CTGATGGAGTATGGTGGAGG-3′
rs2069849	7:22731537	Downstream variant 500B, missense, synonymous codon	*BsrDI*	C/ T	F: 5′-CGTGCATGACTTCAGCTTTACT-3′ R: 5′-CAGTGACCAGATTAACAGGCTAGA-3′

### Statistical analysis

Data was analyzed by the SPSS 22.0 software (SPSS Inc, Chicago), and mean and standard deviation (SD) of the normal distribution continuous variables were calculated and compared using the Student’s *t* test. The categorical variables and percentages were tested using the chi-squared test. The Hardy-Weinberg equilibrium test in the control was performed using SNPstats (http://bioinfo.iconcologia.net/SNPstats). Generalized multi-factor dimension reduction (GMDR) is used to identify the optimal linkage between four SNPs and OST risk. SHEsis software evaluated the haplotype, and logistic regression was performed to assess the association between four SNPs within IL-6 gene and the risk of OST. Two-tailed *P* values < 0.05 were set as statistically significant.

## Results

A total of 1524 postmenopausal females consisted of 758 osteoporosis patients and 766 normal controls were included in this study. The mean age of all participants was 66.2 ± 15.6 years, and there was no difference between case and control group in terms of age. Participants’ characteristics stratified by OST cases and controls were shown in Table 2. The distributions of smoking, alcohol consumption, and mean of BMI and WC were significantly different between cases and controls. The means of lumbar BMD and femoral BMD were significantly lower in OST patients than that in controls.

**Table 2:** General characteristics of study participants in case and control group

Variables	Osteoporosis cases (*n* = 758)	Controls (*n* = 766)	*P* values
Age (years) (means ± SD)	65.5 ± 16.1	66.7 ± 17.0	0.157
BMI (kg/m^2^) (means ± SD)	24.4 ± 5.9	23.3 ± 5.6	0.0002
WC (cm) (means ± SD)	86.5 ± 16.1	84.2 ± 15.6	0.005
Smoke, *N* (%)	62 (8.2)	39 (5.1)	0.015
Alcohol drinking, *N* (%)	102 (13.5)	60 (7.8)	0.0004
Calcium supplementation, *n* (%)	244 (32.2)	223 (29.1)	0.192
YSM (years) (means ± SD)	9.52 ± 5.84	10.03 ± 5.65	0.083
*T* score			
Femoral neck	− 2.73 ± 0.69	0.02 ± 0.30	0.000
Total femoral	− 1.57 ± 1.01	0.03 ± 0.24	0.000
Lumbar spine	− 0.75 ± 0.91	0.45 ± 0.82	0.000
Lumbar BMD (g/cm^2^) (means ± SD)	0.90 ± 0.14	1.05 ± 0.13	0.000
Femoral BMD (g/cm^2^) (means ± SD)	0.82 ± 0.10	0.95 ± 0.11	0.000

*YSM* years since menopause; *BMI* body mass index; *WC* waist circumference; *BMD* bone mineral density

All genotypes were distributed according to Hardy–Weinberg equilibrium (all *P* values were more than 0.05) (Table 3). The frequencies of G allele of rs1800796 and the T allele of rs2069849 were higher in individuals with OST compared to healthy controls (30.5% of OST patients and 22.1% of controls *P* < 0.001 for G allele of rs1800796; 29.2% of OST patients and 20.9% of controls *P* < 0.001 for the T allele of rs2069849). Logistic regression model revealed that G allele of rs1800796 and the T allele of rs2069849 were associated with increased OST risk, compared to those with wild genotype. However, no significant correlations were found when analyzing the association of rs1800795 and rs1554606 with OST risk.

**Table 3:** The association of genotype and allele within four SNPs with OP risk

SNPs	Genotypes and alleles	Frequencies *N*(%)		*OR (95% CI)* ^*^	*HWE test*
		OP patients (*n* = 758)	Controls (*n* = 766)		
rs1800796-572C/G	CC	377 (49.7)	469 (61.2)	1.00	0.346
	CG	300 (39.6)	255 (33.3)	1.39 (1.09–1.65)	
	GG	81 (10.7)	42 (5.5)	1.90 (1.29–2.54)	
	CG + GG	381 (50.3)	297 (38.8)	1.47 (1.13–1.78)	
	C	1054 (69.5)	1193 (77.9)		
	G	462 (30.5)	339 (22.1)		
rs1800795-174G/C	GG	399 (52.6)	440 (57.4)	1.00	0.106
	GC	285 (37.6)	270 (35.2)	1.07 (0.94–1.39)	
	CC	74 (9.8)	56 (7.3)	1.12 (0.85–1.60)	
	GC + CC	359 (47.4)	326 (42.6)	1.09 (0.91–1.44)	
	G	1083 (71.4)	1150 (75.1)		
	C	433 (28.6)	382 (24.9)		
rs2069849	CC	384 (50.7)	481 (62.8)	1.00	0.730
	CT	306 (40.4)	250 (32.6)	1.65 (1.38–1.90)	
	TT	68 (9.0)	35 (4.6)	2.03 (1.63–2.84)	
	CT + TT	374 (49.3)	285 (37.2)	1.74 (1.46–2.05)	
	C	1074 (70.8)	1212 (79.1)		
	T	442 (29.2)	320 (20.9)		
rs1554606 (185C/A)	CC	405 (53.4)	447 (58.4)	1.00	0.272
	CA	292 (38.5)	269 (35.1)	1.08 (0.91–1.36)	
	AA	61 (8.1)	50 (6.5)	1.04 (0.82–1.53)	
	CA + AA	353 (46.6)	319 (41.6)	1.07 (0.89–1.39)	
	C	1102 (72.7)	1163 (75.9)		
	A	414 (27.3)	369 (24.1)		

^*^Adjusted for age, smoke, obesity, and alcohol consumption status.

We employed the GMDR analysis to assess the impact of the IL-6 gene–environment interaction on OST risk, after adjustment for covariates (Table 4). The environment factors including smoking, obesity, and alcohol drinking were included in the GMDR model one by one, and the results suggested that the interaction model composed of the rs1800796 and obesity, was the best model with statistical significance (*P* value from sign test [*P*_sign_] = 0.012), indicating a potential gene–environment interaction between rs1800796 and obesity. Overall, the two-locus models had a cross-validation consistency of 10/10 and had the testing accuracy of 0.641. In order to obtain the odds ratios and 95% CI for the joint effects of rs1800796 genotype and obesity on OST, we conducted stratified analysis for rs1800796 genotype and obesity and found that obese subjects with CG or GG genotype have the highest OST risk, compared to subjects with CC genotype and normal BMI OR (95% CI) = 2.21 (1.52–3.49), after adjustment for age, smoke, and alcohol consumption status (Table 5).

**Table 4:** Best gene–environment interaction models as identified by GMDR

Locus no.	Best combination	Cross-validation consistency	Testing accuracy	*P* values^*^
Gene–alcohol drinking interaction*				
2	1, 5	6/10	0.522	0.324
3	1, 3, 5	7/10	0.514	0.255
4	1, 2, 3, 5	6/10	0.597	0.212
5	1, 2, 3, 4, 5	7/10	0.523	0.240
Gene–smoking interaction**				
2	3, 6	7/10	0.488	0.536
3	3, 1, 6	6/10	0.523	0.706
4	1, 2, 3, 6	5/10	0.536	0.862
5	1, 2, 3, 4, 6	6/10	0.518	0.377
Gene–obesity interaction***				
*2*	*1, 7*	*10/10*	*0.641*	*0.012*
3	1, 2,7	8/10	0.526	0.266
4	1, 2, 4, 7	9/10	0.514	0.545
5	1, 2, 3, 4, 7	5/10	0.634	0.372

^*^Adjusted for age, smoke, and obesity

^**^Adjusted for age, alcohol consumption status, and obesity

^***^Adjusted for age, smoke, and alcohol consumption status

Numbers 1–7 represent *rs1800796*, rs1800795, *rs2069849*, rs1554606, alcohol drinking, smoking, and obesity, respectively

**Table 5:** Stratified analysis for interaction between *rs1800796* and obesity using logistic regression

*rs1800796*	Obesity	OR (95% CI) ^*^	*P* values
CC	No	1.00	–
CG or GG	No	1.21 (1.05–1.52)	0.030
CC	Yes	1.43 (1.10–1.85)	0.003
CG or GG	Yes	2.21 (1.52–3.49)	< 0.001

^*^Adjusted for age, smoke, and alcohol consumption status

## Discussion

Our study showed that both alleles of G allele of rs1800796 and the T allele of rs2069849 were correlated with the increased OST risk in Chinese postmenopausal women. But we did not identify any relationship between rs1800795 and rs1554606 genotype or alleles with OST risk in this population. Our research is not the first case-control study for the relationship between SNP in IL-6 genes and OST risk, and several studies have been conducted to investigate this relationship previously. However, the previous data were conflicting and had no conclusive. The results of a previous study [16] suggested an association of the IL-6 -174 G/C polymorphism with osteoporosis in postmenopausal women in a Polish population. Deveci et al. [17] indicated that IL-6 promoter region SNP showed an association with BMD in this postmenopausal Turkish population, and these data suggest that the wild GG genotype influences the phenotype. Blumenfeld et al. [18] supported the conjecture that inflammation mediated by variation in the IL-6 genomic region is associated with hand osteoarthritis and osteoporosis-related phenotypes. A Chinese study concluded that IL-6 polymorphisms were associated with overall and site-specific OST in both sexes, but this association was dependent on the genotypes of the leptin receptor (LEPR). Previous, Ota et al. [19] and Garnero et al. [9] indicated that polymorphisms at the rs1800795 and rs1800796 positions in the promoter region were associated with BMD in post-menopausal women. The epidemiological data suggest that circulating IL-6 is a significant factor in bone loss [20], as well as in the development of osteoarthritis-related phenotypes [21]. However, inconsistent results also existed. Garnero et al. [9] found no significant association between genotypes, bone turnover markers in premenopausal women, and either bone turnover or BMD in postmenopausal women. Walston et al. [22] suggested that IL-6 gene variation may not be an important factor in the determination of elevated IL-6 levels and related phenotypes found in older women. The mechanism on the association between IL-6 and OST was not well known yet. Previous study [23] has indicated that inflammation as a contributing factor among postmenopausal women with osteoporosis. Cytokines as IL-6, which potently induces osteoclastogenesis, is produced by osteoblastic cells in response to parathyroid hormone (PTH) [24], PTH could indirectly stimulate bone resorption by acting upon its receptors on osteoblasts. IL-6 also appears to be one of the central mediators of osteoclast activity, activates bone resorption pathways, supporting a crucial role in osteoporosis [25]. In this study, the plasma concentrations of IL-6 were not obtained for all participants, however, the study also suggested that the minor allele of IL-6 gene was also found to be associated with lower plasma concentrations of IL-6 in healthy individuals [26].

The multi-phenotypes of OST were the result of many genetic polymorphism and gene–environment interactions, in addition, the inconsistency of the previous studies in the genetic risks involved in the proposed osteoporosis may be attributed to the results of population mixing, gene–environment interactions. In recent years, some studies indicated that obesity was a risk factor for OST [12, 13], and some study also suggested smoking [14] and alcohol drinking [15] were associated with OST. In this study, the rate for obesity, smoking, and drinking were all higher in OST patients. So these environmental factors were included in the GMDR model, which showed a significant rs1800796–obesity interaction, but no significant interaction between SNPs and smoking or alcohol drinking was noted. Our data suggest that the risk of osteoporosis may be altered by the obesity status and that their susceptibility to osteoporosis risk interacts. However, our study did not confirm any interaction combinations between SNP and smoking or alcohol drinking associated with osteoporosis risk. The detailed mechanism for rs1800796–obesity interaction was not well known; however, obesity was also an important risk factor for OST, and we hypothesized that an interplay effect existed between rs1800796 and obesity in the progression for OST and because both rs1800796 and obesity were related with inflammation and were risk factors for OST.

The limitations of our research are (1) studies with relatively small sample sizes and large sample sizes should be further used to confirm this association. (2) The study should include more SNPs within the IL-6 gene. (3) More environment factors should be included in the gene–environment interaction analysis.

## Conclusion

The G allele of rs1800796 and the T allele of rs2069849, interaction between rs1800796 and obesity, were correlated with the increased OST risk in Chinese postmenopausal women.

## Data Availability

Not applicable

## Abbreviations

GMDR: Generalized multifactor dimensionality reduction
IL-6: Interleukin-6
OST: Osteoporosis
PCR-RFLP: Polymerase chain reaction-based restriction fragment length polymorphism
SNP: Single-nucleotide polymorphisms

## References

[1] Zwart M, Azagra R, Encabo G, Aguye A, Roca G, Guell S, Puchol N, Gene E, Lopez-Exposito F, Sola S, Ortiz S, Sancho P, Abado L, Iglesias M, Toran P, Diez-Perez A, Pujol-Salud J (2011). Measuring health-related quality of life in men with osteoporosis or osteoporotic fracture. BMC Public Health.

[2] Kelly PJ, Morrison NA, Sambrook PN, Nguyen TV, Eisman JA (1995). Genetic influences on bone turnover, bone density and fracture. Eur J Endocrinol.

[3] Liu Z, Zhao Y, Ding G, Zhou Y (1997). Epidemiology of primary osteoporosis in China. Osteoporos Int.

[4] Rizzoli R, Bonjour JP, Ferrari SL (2001). Osteoporosis, genetics and hormones. J Mol Endocrinol.

[5] Peacock M, Turner CH, Econs MJ (2002). Genetics of osteoporosis. Endocr Rev.

[6] Yamaguchi N, Suzuki Y, Mahbub MH, Takahashi H, Hase R, Ishimaru Y, Sunagawa H, Watanabe R, Eishi Y, Tanabe T (2017). The different roles of innate immune receptors in inflammation and carcinogenesis between races. Environ Health Prev Med.

[7] Mao SQ, Sun JH, Gu TL, Zhu FB, Yin FY, Zhang LN (2017). Hypomethylation of interleukin-6 (IL-6) gene increases the risk of essential hypertension: a matched case–control study. J Hum Hypertens.

[8] Yan L, Hu R, Tu S, Cheng WJ, Zheng Q, Wang JW, Kan WS, Ren YJ (2015). Meta-analysis of association between IL-6 -634C/G polymorphism and osteoporosis. Genet Mol Res.

[9] Garnero P, Borel O, Sornay-Rendu E, Duboeuf F, Jeffery R, Woo P, Delmas PD (2002). Association between a functional interleukin-6 gene polymorphism and peak bone mineral density and postmenopausal bone loss in women: the OFELY study. Bone.

[10] Valdmanis PN, Kabashi E, Dion PA, Rouleau GA (2008). ALS predisposition modifiers: knock NOX, who's there? SOD1 mice still are. Eur J Hum Genet.

[11] Uitterlinden AG, van Leeuwen JP, Pols HA, Marcus R, Feldman D, Kelsey J (2001). Genetics and genomics of osteoporosis. Osteoporosis.

[12] Neglia Cosimo, Argentiero Alberto, Chitano Giovanna, Agnello Nadia, Ciccarese Roberta, Vigilanza Antonella, Pantile Valerio, Argentiero Domenico, Quarta Raffaele, Rivezzi Matteo, Di Tanna Gian, Di Somma Carolina, Migliore Alberto, Iolascon Giovanni, Gimigliano Francesca, Distante Alessandro, Piscitelli Prisco (2016). Diabetes and Obesity as Independent Risk Factors for Osteoporosis: Updated Results from the ROIS/EMEROS Registry in a Population of Five Thousand Post-Menopausal Women Living in a Region Characterized by Heavy Environmental Pressure. International Journal of Environmental Research and Public Health.

[13] Fassio A, Idolazzi L, Rossini M, Gatti D, Adami G, Giollo A, Viapiana O (2018). The obesity paradox and osteoporosis. Eat Weight Disord.

[14] Chen H, Liu N, Xu X, Qu X, Lu E (2013). Smoking, radiotherapy, diabetes and osteoporosis as risk factors for dental implant failure: a meta-analysis. PLoS One.

[15] Abukhadir SS, Mohamed N, Mohamed N (2013). Pathogenesis of alcohol-induced osteoporosis and its treatment: a review. Curr Drug Targets.

[16] Czerny B, Kaminski A, Kurzawski M, Kotrych D, Safranow K, Dziedziejko V, Bohatyrewicz A, Pawlik A (2010). The association of IL-1b, IL-2, and IL-6 gene polymorphisms with bone mineral density and osteoporosis in postmenopausal women. Eur J Obstet Gynecol Reprod Biol.

[17] Deveci D, Ozkan ZS, Yuce H (2012). Is there any relation between IL-6 gene -174 G>C polymorphism and postmenopausal osteoporosis?. Eur J Obstet Gynecol Reprod Biol.

[18] Lin CC, Li TC, Liu CS, Yang CW, Lin CH, Hsiao JH, Meng NH, Lin WY, Liao LN, Li CI, Wu FY (2016). Associations of TNF-a and IL-6 polymorphisms with osteoporosis through joint effects and interactions with LEPR gene in Taiwan: Taichung Community Health Study for Elders (TCHS-E). Mol Biol Rep.

[19] Ota N, Nakajima T, Nakazawa I, Suzuki T, Hosoi T, Orimo H, Inoue S, Shirai Y, Emi M (2001). A nucleotide variant in the promoter region of the interleukin-6 gene associated with decreased bone mineral density. J Hum Genet.

[20] Scheidt-Nave C, Bismar H, Leidg-Bruckner G, Woitge H, Seibel MJ, Ziegler R, Pfeilschifter J (2001). Serum Interleukin 6 Is a Major Predictor of Bone Loss in Women Specific to the First Decade Past Menopause. J Clin Endocrin Metabol.

[21] Livshits G, Zhai G, Hart DJ, Kato BS, Wang H, Williams FM, Spector TD (2009). Interleukin-6 is a significant predictor of radiographic knee osteoarthritis: the Chingford Study. Arthritis Rheum.

[22] Walston J, Arking DE, Fallin D, Li T, Beamer B, Xue Q, Ferrucci L, Fried LP, Chakravarti A (2005). IL-6 gene variation is not associated with increased serum levels of IL-6, muscle, weakness, or frailty in older women. Exp Gerontol.

[23] Al-Daghri NM, Aziz I, Yakout S, Aljohani NJ, Al-Saleh Y, Amer OE, Sheshah E, Younis GZ, Al-Badr FB (2017). Inflammation as a contributing factor among postmenopausal Saudi women with osteoporosis. Medicine (Baltimore).

[24] Masiukiewicz US, Mitnick M, Grey AB, Insogna KL (1999). A role for interleukin-6 in parathyroid hormone-induced bone resorption in vivo 1. Endocrinology.

[25] Edwards CJ, Williams E (2010). The role of interleukin-6 in rheumatoid arthritis-associated osteoporosis. Osteoporos Int.

[26] Fishman D, Faulds G, Jeffery R, Mohamed-Ali V, Yudkin JS, Humphries S, Woo P (1998). The effect of novel polymorphism in the interleukin 6 gene on IL-6 transcription and plasma IL-6 levels, and an association with systemic-onset juvenile chronic arthritis. J Clin Invest.

